# T-Cell Non-Hodgkin Lymphoma of the Ileum Presenting as Perforation and Peritonitis: A Case Report

**DOI:** 10.3389/fsurg.2022.810360

**Published:** 2022-03-15

**Authors:** Yong Tian, Chuanfang Li, Rusong Tian, Qingming Tian, Song Qiao

**Affiliations:** Department of Gastrointestinal Surgery, Tongren City People's Hospital, Tongren, China

**Keywords:** T-cell lymphoma, intestinal perforation, peritonitis, acute abdominal syndrome, case report

## Abstract

**Background:**

Non-Hodgkin lymphoma (NHL) of the ileum, presenting as perforation and peritonitis, is a rare disease, derived from intestinal intraepithelial T lymphocytes. The degree of malignancy is extremely high. The pathogenesis of ileal perforation caused by NHL remains unclear, as well as the chromosome and immune system abnormalities, which may be related to NHL, and are indistinguishable from other benign and malignant conditions and are clinically nonspecific.

**Case Report:**

We describe an 84-year-old man with abdominal pain for 4 days, which was aggravated for 3 h. The pain was in the upper abdominal region and was initially considered to be due to gastrointestinal perforation. He had persistent insidious pain, accompanied by nausea, vomiting, and fever. Physical examination indicated that the patient had pain all over the abdomen; also, rebound pain and muscle tension, and bowel sounds were reduced on auscultation. An abdominal CT scan showed free gas in the abdominal cavity. The patient was diagnosed with peritonitis due to hollow viscus perforation. A prompt exploratory laparotomy was performed. Intraoperative findings showed perforations in the ileum that are approximately 40 cm from the ileocecal region, which were 3–8 mm in size. A segmental distribution was observed, and the intestinal contents overflowed with purulent discharge around the perforation surface. Resection and ileostomy were performed, and the clinical histopathological examination confirmed T-cell lymphoma. The patient was advised to visit the Oncology Department for further chemotherapy.

**Conclusion:**

Timely emergency surgery is the key to the treatment of ileal perforation caused by T-cell lymphoma. Resection and ileostomy were performed as intervention measures, and subsequent histopathological examination manifested T-cell lymphoma.

## Introduction

Non-Hodgkin lymphoma (NHL) of the ileum is an intestinal tumor of intraepithelial T-lymphocytes, usually presenting as a neoplasm composed of large lymphoid cells and is associated with necrosis and inflammatory background, as well as the presence of large numbers of histiocytes and eosinophils (1). The small intestinal perforation is caused by the primary T-cell lymphoma, which is an extremely rare disease that is strongly related to celiac disease. A primary T-cell lymphoma is associated with a very poor prognosis and a high mortality rate (2).

In our daily clinical practice, most acute abdominal syndromes are characterized by pain, which requires an early diagnosis and treatment, while most require emergency surgery. Common acute abdominal diseases include appendicitis, gastric and intestinal perforation, volvulus, acute pancreatitis, and ectopic pregnancy. Therefore, in most cases, the intestinal perforation can only be detected by a laparotomy, and a definite diagnosis can only be made after a histopathological examination. We report the rare case of a male suffering from peritonitis with a non-Hodgkin lymphoma located in the small intestine. This case is reported in accordance with the SCARE 2018 guidelines (3).

The 84-year-old male patient has an uncommon case of suffering from perforation and peritonitis, with a T-cell lymphoma located in the small intestine and treated in Tongren People's Hospital in September 2020. The medical history, clinical symptoms, signs, laboratory results, imaging data, and histopathological examination results were reported as follows.

## Case Report

An 84-year-old male patient was presented to the hospital with abdominal pain for 4 days, which was aggravated for 3 h. The pain was initially located in the upper abdominal area and was a persistent insidious pain. The patient felt unbearable pain accompanied by nausea, vomiting, and fever due to the gradual deterioration of the disease. The patient had a history of valvular heart disease and coronary atherosclerosis for 10 years, but no history of peptic diseases or black feces, weight and appetite loss, hepatitis, tuberculosis, typhoid, smoking, and alcohol consumption.

He does not have a history of familial hereditary disease. A clinical examination revealed a heart rate of 132 beats per minute, respiratory rate of 22 breaths per minute, blood pressure of 130/80 mmHg, and temperature of 36.5°C. A physical examination indicated that the patient had pain all over the abdomen, rebound pain, and muscle tension. The liver and spleen were not palpable, and the percussion demonstrated a drumming sound around the umbilicus. The shifting dullness was negative, and bowel sounds were reduced on auscultation. Other systems were normal. There were no palpable lymph nodes in the cervical, axillary, or inguinal regions.

**Figure 1:**
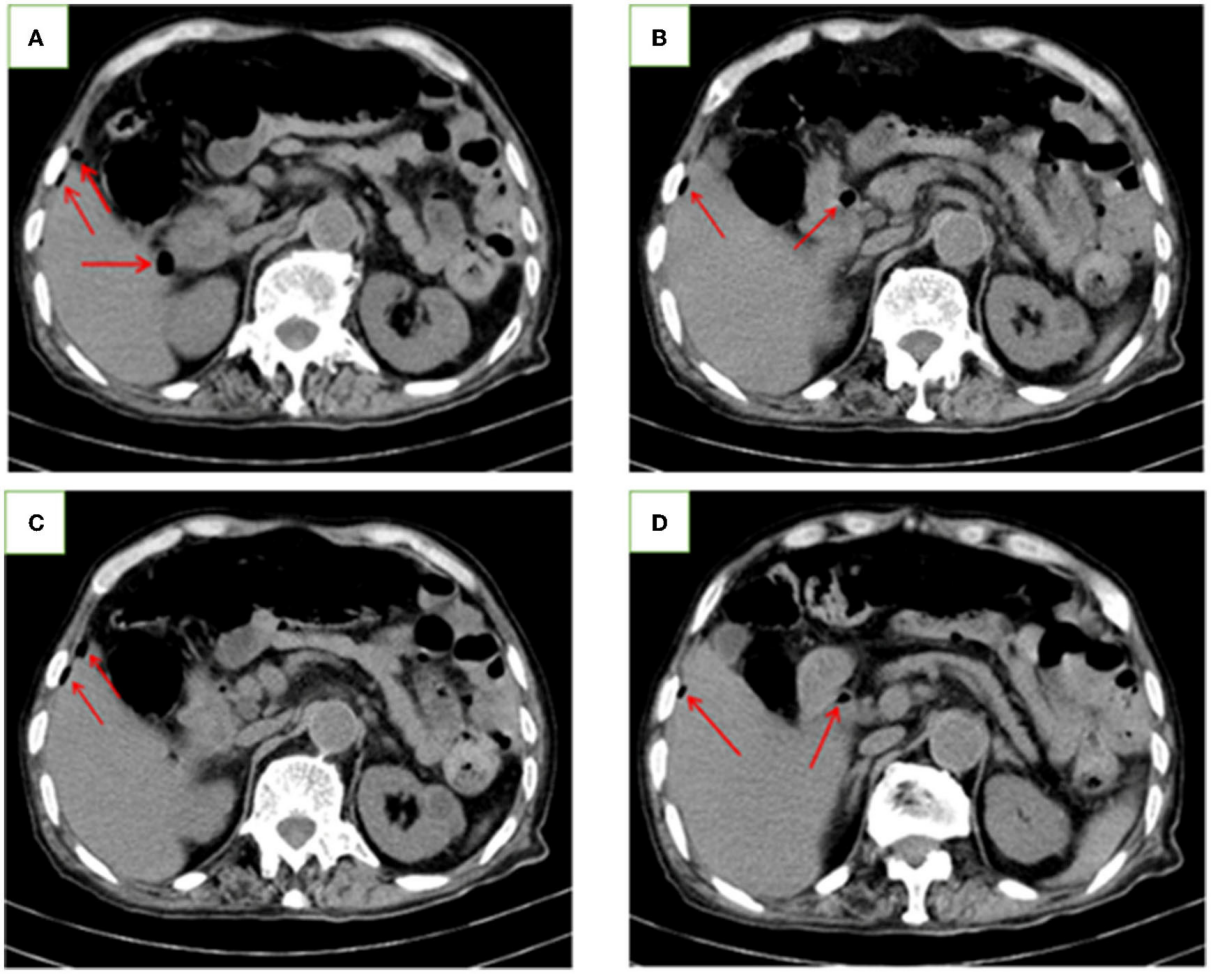
Abdominal CT images of the patient. **(A–D)** Abdominal CT scan showing the free gas (red arrow) in the abdominal cavity.

The complete blood count indicated a leukocyte count of 20.01 × 10^9^, with a neutrophil composition of 94.4%, and hemoglobin content of 86 g/L. The biochemical indices were within the normal limits. C-reactive protein content was 255.55 mg/L. The coagulation test was normal. The patient was negative for hepatitis B, syphilis, and HIV. The electrocardiogram and chest X-ray were also normal. An abdominal CT scan revealed a free gas in the abdominal cavity (Figure 1). The patient was diagnosed with peritonitis due to the perforation of the hollow viscus.

**Figure 2:**
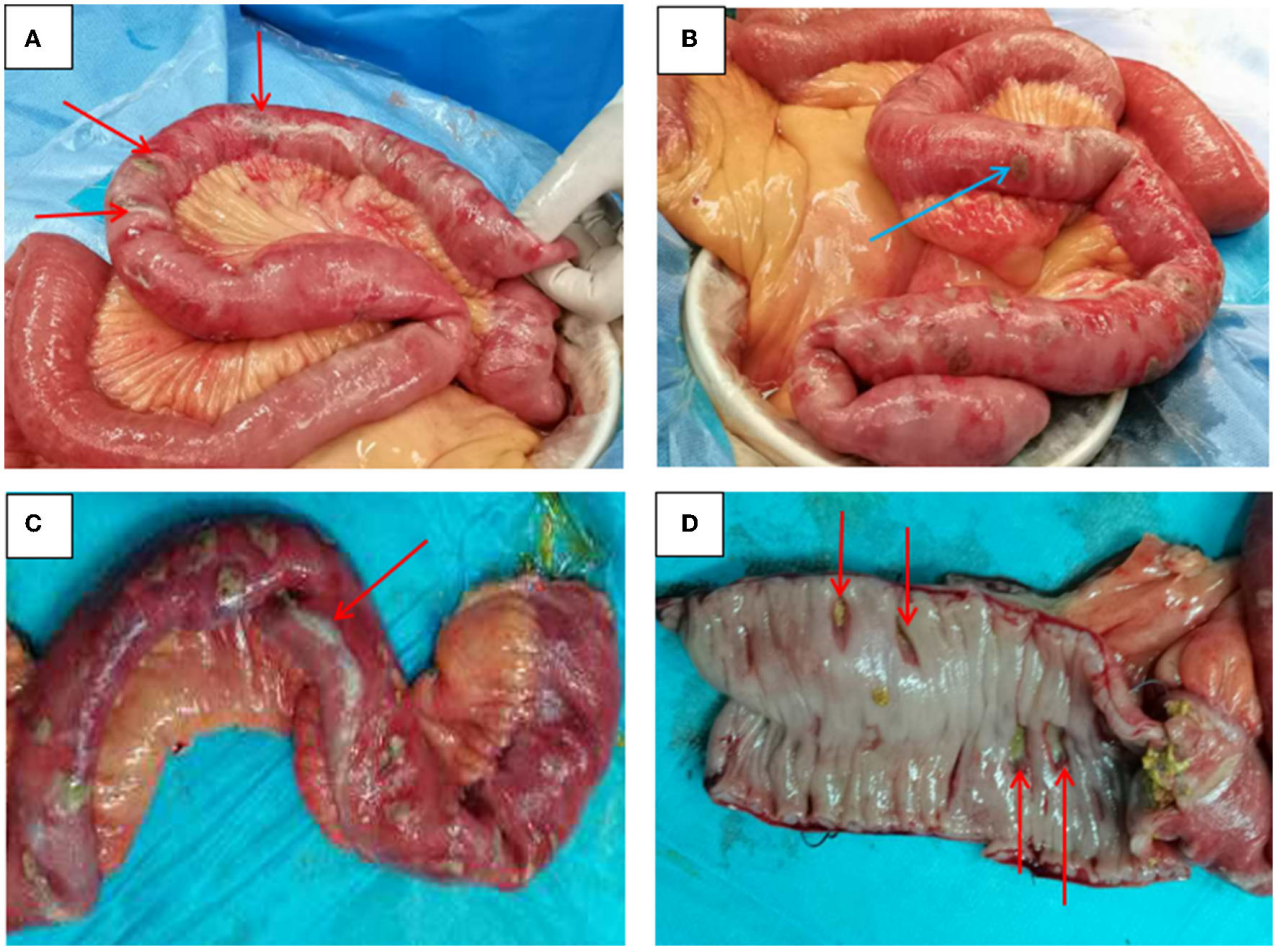
Intraoperative findings. **(A)** Ileum perforation (red arrow) with no discreet mass lesion; **(B)** Perforation of the ileum ~30 cm from the ileocecal region and about 40 cm in size (blue arrow); **(C)** A large amount of purulent discharge (red arrow) was found in the intestinal wall and the perforation focus; **(D)** Ulcers (red arrow) were seen in the ileum mucosa and have penetrated different levels, including the full thickness of the bowel wall.

After a relevant preoperative preparation, an exploratory laparotomy was immediately performed using the right *rectus abdominis* incision and the abdomen was opened in layers. Approximately 400 ml of bowel content was found in the abdominal cavity. There were perforations in the ileum that was ~40 cm from the ileocecal region, which were 3–8 mm in size, a segmental distribution was observed, and the intestinal contents had overflowed with purulent moss around the perforation surface (Figure 2). No perforation or tumor was observed in the stomach, liver, spleen, and peritoneum. According to the intraoperative findings, we considered that the small intestinal perforation may have been caused by Crohn's disease, intestinal tuberculosis, or ileotyphus.

**Figure 3:**
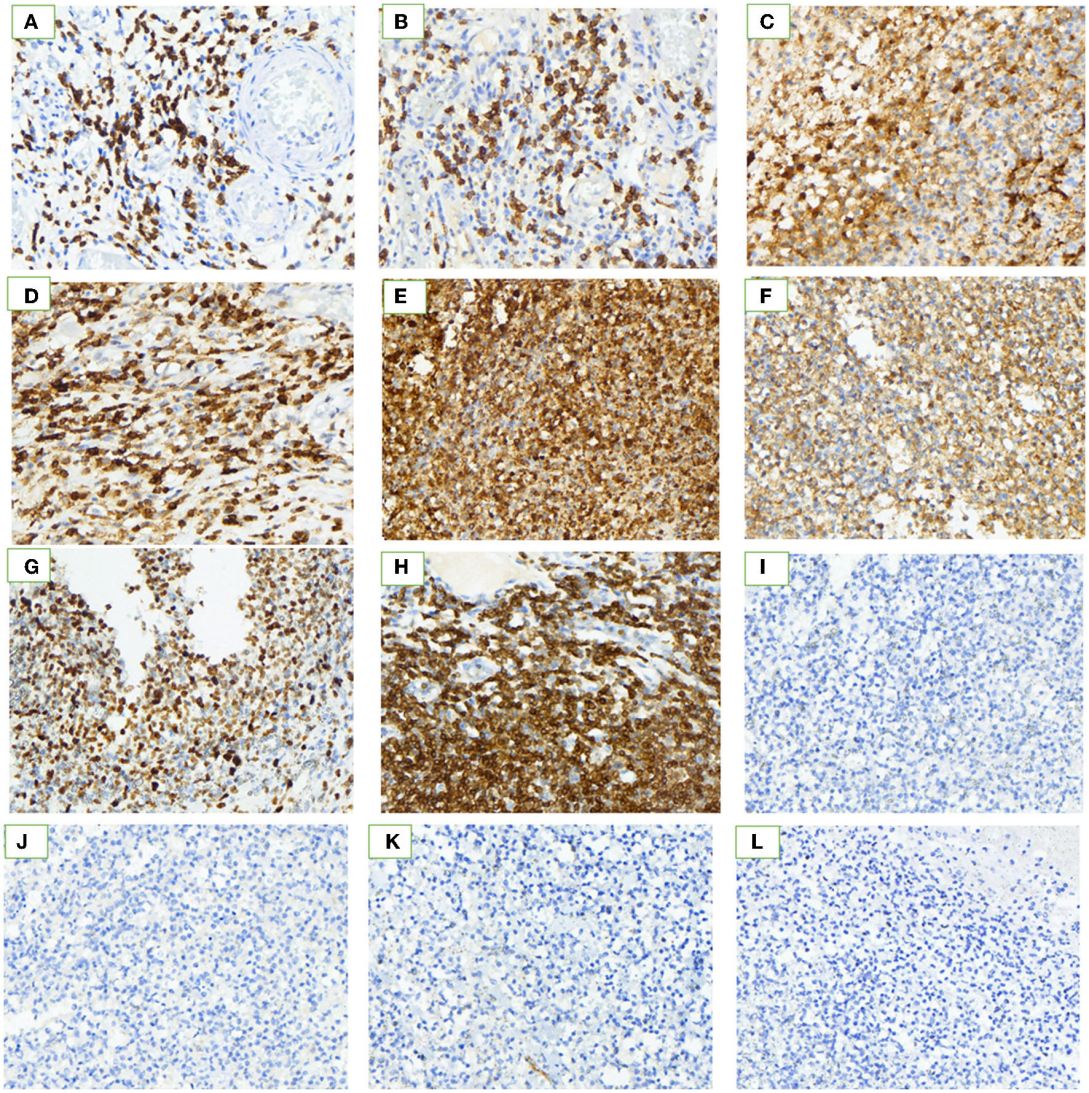
Immunohistochemistry. Histopathological examination showed hematoxylin and eosin stains (H&E × 40). The neoplastic lymphocytes are small-to-medium-size with dark staining nuclei, conspicuous nucleoli, and scarce cytoplasm, and have infiltrated all the layers of the jejunal wall. The immunohistochemical characteristics of tumor cells. **(A–I)** Immunohistochemical stain is strongly positive for the neoplastic cells. **(J–L)** Immunohistochemical stain is negative. Positive: **(A)** CD3; **(B)** CD5; **(C)** CD10; **(D)** CD45R0; **(E)** CD15; **(F)** TIA-1; **(G)** Ki-67 (40%); **(H)** Bcl-2; and **(I)** CD138. Negative: **(J)** Cyclin D1; **(K)** CD56; **(L)** CD30.

We performed a resection of the ileum segment and its mesentery, and an ileostomy in the left lower abdomen. The resected ileum specimen was sent to the Pathology Department for analysis. Ten days post-operation, the histopathological examination of the specimens revealed the features of malignant NHL of the T-cell type, and immunohistochemistry-staining showed that the leucocyte antigen was positive (CD45R0, Bcl-2, CD3, CD5, CD10, CD15, Ki-67, CD138, TIA-1, and CKi67 [40%]) on the tumor cells, and negative CyclinD1, CD56, and CD30 were observed (Figure 3), indicating that the lesion was of a T-cell origin, rather than a B-cell origin or a Hodgkin lymphoma type.

The patient was discharged postoperatively in 8 days in good condition, and had a good spirit and diet, without any special discomfort. The patient was advised to visit the Oncology Department for further chemoradiotherapy.

## Discussion

An acute abdomen signifies the need for prompt diagnosis and early treatment, but not necessarily a surgical treatment all the time; the pain is the main symptom in most patients (4).

In the clinical work, perforation of the digestive tract caused by lymphoma is uncommon, and the small intestine (particularly the proximal jejunum) is the most common site of involvement followed by the stomach, colon, and rectum (5, 6). A small intestinal lymphoma is predominantly found in the ileum (60–65%), followed by the jejunum (20–25%), duodenum (6–8%), and other sites (8–9%) (7).

Previous research reported that the primary intestinal lymphoma is a predominantly male disease, the male:female ratio is 2.5:1. The clinical presentation of small intestinal lymphoma is non-specific, and the patients mainly have acute abdominal pain symptoms (70–80%), weight loss (30%), hematochezia (25.9%), diarrhea (16.9%) nausea, vomiting, and bad appetite (8–10), but rarely a perforation and acute obstructive symptoms (11). The non-specific clinical manifestations of NHL make the preoperative diagnosis extremely difficult, and the diagnosis can only be confirmed by a postoperative pathological examination.

We report the case of an 84-year-old male patient with a primary ileum T-cell lymphoma. The first symptom was acute abdominal pain, which has gradually manifested as peritonitis. We excluded relevant contraindications and identified numerous perforations in the ileum by performing an emergency laparotomy, which required an ileum resection and ileostomy. We conclude that a temporary enterostomy may be a better choice for patients with unexplained spontaneous intestinal perforation, rather than an intestinal resection and intestinal anastomosis.

Given its rarity and its low incidence rate, the intestinal perforation caused by lymphoma is often neglected and is difficult to distinguish from Crohn's disease, intestinal tuberculosis, intestinal typhoid, and other types of gastrointestinal disease. For patients with abdominal pain, fever, gastrointestinal perforation, and bloody stool, especially when multiple ulcers of the intestinal wall are observed during colonoscopy, the possibility of lymphoma should be considered to avoid misdiagnosis or missed diagnosis (12). Therefore, gastrointestinal surgeons should improve their current understanding of the disease, especially our young doctors. Timely intervention and good clinical management will yield favorable results.

Surgery followed by chemotherapy is the recommended treatment and surgery followed by an adjuvant multi-agent chemotherapy, with cyclophosphamide, doxorubicin, vincristine, and prednisolone (CHOP), has led to improved outcomes. In most cases, the NHL is usually treated with anthracycline-based therapy consisting of CHOP (13).

In conclusion, the prognosis of patients with NHL remains exceedingly poor (14, 15). The results in an extremely variable 5-year survival of patients with NHL are between 8 and 60% (16, 17).

## Follow-Up

We followed up with the patient by telephone every month. He received 5 cycles of postoperative chemotherapy. At present, he has a good mental state and diet, no recurrence of tumor, no cough, expectoration, no abdominal pain, distention, fever, or other symptoms. There were no signs of recurrence on the physical examination and the CT scan, with contrast enhancement of the chest, abdomen, and pelvis (C/A/P CT Scan), was within the normal limits and was carried out every 3–6 months for the first 5 years, then, every 12 months for each subsequent year. We were unable to perform the PET as this technique is not available in our hospital.

## Conclusion

The clinical presentation of a primary small intestinal lymphoma is usually nonspecific, which easily leads to a delayed diagnosis and a misdiagnosis. Early diagnosis and treatment are important for improving the prognosis of bowel perforation in patients with NHL. A surgery followed by chemotherapy is the recommended treatment for improving the overall survival of patients.

## Data Availability Statement

The original contributions presented in the study are included in the article/supplementary material, further inquiries can be directed to the corresponding author/s.

## Ethics Statement

Written informed consent was obtained from the participant and the study protocol was approved by the Research Ethics Board of the Tongren City People's Hospital, Tongren City, Guizhou Province, China.

## Author Contributions

YT studied the literature and wrote the manuscript. CL, QT, and RT operated on the patient and had the idea for this case report. SQ checked the manuscript and made the corrections. All authors read and approved the final manuscript.

## Conflict of Interest

The authors declare that the research was conducted in the absence of any commercial or financial relationships that could be construed as a potential conflict of interest.

## Publisher's Note

All claims expressed in this article are solely those of the authors and do not necessarily represent those of their affiliated organizations, or those of the publisher, the editors and the reviewers. Any product that may be evaluated in this article, or claim that may be made by its manufacturer, is not guaranteed or endorsed by the publisher.
